# Pott's Fracture and Its Allies

**Published:** 1903-05-16

**Authors:** 


### HOSPITAL CLINICS AND MEDICAL PROGRESS.

### POTT'S FRACTURE AND ITS ALLIES.

### GENERAL ANÆSTHESIA AND MASSAGE.—THE VALUE OF X-RAYS.

SOME of the most troublesome injuries from the point of view of treatment are those involving the bones and ligaments in the vicinity of the ankle joint. The swelling and pain often make it impossible to ascertain the exact extent and nature of the injury at the time when the patient is first seen. The small size of the broken fragments greatly increases the difficulty of treatment, so that in some cases, in spite of all skill and patience, a certain amount of displacement cannot be altogether overcome, and some deformity results, leaving a joint which is weak and painful, and of which the mobility is seriously impaired. Pott's fracture is the commonest injury of this nature, but there are other fractures closely allied, and though Pott himself did not include these in the same category as that particular one bearing his name, he drew so much attention to the importance of this class of injury that it is convenient to speak of them together. The following varieties are recognised:—(1) The fibula is fractured at its weakest part, 2 to 3 inches above the ankle, the internal lateral ligament being torn through. (2) The fibula is fractured 3 inches above the ankle, and the tip of the internal malleolus is torn off. (3) The fibula is fractured 3 inches above the ankle, and the lower end of the tibia is splintered obliquely downwards. (4) The internal malleolus alone is torn off, the fibula remaining sound. A common displacement is one in which the foot is displaced outwards and the sole is everted, the heel being drawn up, the toes pointing downwards and outwards, and the inner border of the sole becoming the part on which the patient would rest were he able to stand.

While in some cases there may be no displacement, in others it is so extreme that the foot is rotated through a right angle to its normal axis, and the toes point directly outwards, and the heel directly inwards. Between these two conditions all grades of displacement may be met with and need to be overcome. Though some cases do perfectly well, the occasional disappointing results too frequently seen even when there is no external wound, are common to all four varieties. The reason is that the internal lateral ligament of the ankle-joint is so firmly attached to the tibia on the one hand, and to the tarsal bones on the other, that when torn a portion of the internal malleolus is nearly always torn off with it; thus Pott's fracture and its allies are really fractures into the ankle-joint, and the injury to the shaft of the fibula is merely incidental. Once this is realised, it is easy to comprehend the difficulties and unsatisfactory results that may follow these injuries. Blood is poured into and distends the synovial cavity, fragments of bone or articular cartilage project into the joint, and during the process of repair inflammatory changes taking place around the torn ligament or fractured bones cause adhesions or callus which may permanently encroach upon and diminish the size of the tibio-fibular mortice. In many cases the signs of these fractures are unmistakable, especially when the displacement of the foot is considerable. Often when there is little or no displacement a correct diagnosis can only be arrived at by examination with the $x$-rays, which have proved that many cases of so-called badly sprained ankle are really fractures of the class under consideration. All will allow that to mistake a fracture for a sprain is most undesirable, but that it is possible, even for the most skilful, cannot be denied. An $x$-ray photograph would prevent such error in diagnosis, yet this aid is often not fully appreciated. Where the apparatus is not available one must fall back upon the physical signs, and in these cases examination under an anæsthetic is invaluable. Some sprains are so severe that the extensive laceration of ligament or fascia leads to so much swelling and pain that there may be very real difficulty in determining whether a fracture has not also occurred. Nevertheless rapid onset of swelling, corresponding to the outline of the ankle-joint, coming on after an injury, with tenderness, greatest over the internal malleolus and over that part of the fibula 3 inches above the ankle, even though there be no irregularity of bone, is strongly suggestive of fracture. If compression of the upper half of the tibia and fibula together elicits pain in the lower third of the fibula, especially if the natural spring of the latter is lost, even though there be no crepitus, fracture is almost certainly present. Extensive bruising on both sides of the ankle, extending some distance up the leg, and appearing two or three days after the receipt of the injury, further indicates some deep-seated mischief, especially if there be considerable œdema obscuring the bony points of the ankle.

Before describing the mode of treatment to be adopted, it will be well to inquire into the principles upon which such treatment should be based. A correct anatomical knowledge of the conformation of the bones of this joint is very desirable, and the following points especially may be here considered. The lower extremity and the internal malleolus of the tibia, and the external malleolus of the fibula, form a hollow resembling a mortice, into which the upper surface of the body of the astragalus is received. The joint cavity is wider in front than behind. There is a difference in width of the anterior and posterior parts of the cavity of 0·5 cms. (nearly $\frac{1}{4}$ inch), and there is the same difference in the corresponding breadths of the astragalus. Upon this peculiar shape one of the most important principles of treatment is based. In complete flexion the astragalus is tightly grasped between the two malleoli; when the ankle-joint is fully extended (for instance when standing tip-toe), the narrower posterior part of the astragalus is brought into the broader anterior part of the mortice, and thus a certain amount of lateral movement is allowed. Supposing the foot to be partly extended the fragments of bone, cartilage or fibrous tissue projecting into the joint from the fractured internal malleolus, will narrow the tibio-fibular mortice. This narrowing (unless steps be taken to correct it) will remain permanent, so that the wider anterior part of the astragalus can no longer be received into the narrowed space between the malleoli. This is the explanation of one form of lameness common after these injuries to the ankle. In walking the foot cannot be completely flexed: either the patient must take very short strides, lest he drag the toes, or, as is more usual, he rotates the whole limb outwards, so that the foot points nearly at right angles to the direction of progression. This uncustomary mode of exercise soon becomes irksome, and even with the aid of a stick the patient is easily fatigued and the pain in the ankle is aggravated. What steps can be taken to prevent the narrowing of the intermalleolar space? If in the beginning the foot is well flexed beyond a right angle, so that the widest part of the astragalus is forced into the space between the malleoli, any projecting piece of cartilage or ligament or fragment of bone is pressed aside, and no longer narrows the space into which the astragalus is to be received. To prevent reparative material being thrown into the joint causing narrowing at a later period, this position of full flexion must be maintained (except during massage), until all active process of repair has ceased. At the end of this time, instead of flexion being impaired it will be as free as extension and the patient will walk without lameness or pain.

To prevent a doubtful diagnosis leading to disappointing results, it would be much more satisfactory both to patient and surgeon, if all cases of severe sprain could be examined with the *x*-rays at the earliest convenient occasion; failing that, to place the patient under the influence of a general anæsthetic, and to then examine the injured part, though this latter method is less valuable than an *x*-ray photograph for diagnostic purposes. When a fracture is detected it is safer while the patient is still under the anæsthetic to apply the splints selected. For not only is the patient spared much physical pain, but the surgeon can proceed in his manipulations until he is satisfied that any displacement has been corrected, and the foot is in good position. After setting the fracture, the surgeon should not feel altogether satisfied until a second *x*-ray photograph has been seen; for there is in the worst cases still a possibility of some deformity remaining, and should this pass unrecognised until the splints are finally discarded, the result may be quite the reverse of that anticipated. To prevent disappointment therefore, a second photograph is advisable, taken at the end of a week; especially is this so in a stout and restless patient whose limb is greatly swollen. As regards the selection of splints, the ordinary Neville splint, consisting of a back-splint with rectangular footpiece and sides, is of proved value. The back-splint should reach well above the ham, and the limb should be slung in straps attached to a cradle. If the foot cannot be maintained in a rectangular position, owing to tension of the tendo Achillis, tenotomy may have to be performed, but it is better to make use of that splint known as Roughton's modified Cline. This is an outside wooden splint, hollowed and shaped for the leg, with a rectangular footpiece which should be so padded that the foot is kept fully flexed. By enabling the patient to lie on his side the knee can be fully flexed, thus effecting complete relaxation of the tendo Achillis. By use of Roughton's splint some of the best results of treatment of difficult Pott's fracture and its allies have been obtained. A real advantage is that the patient can be moved early into a chair or on to a couch and so his general health is less likely to suffer than if he be confined entirely to bed with box-splint and cradle. The simplest form of massage, commencing about the fifth day and continued daily until all œdematous swelling has been made to disappear, is most agreeable to the patient. Indeed the value of massage in these cases is inestimable and it is a valuable aid in the process of repair. It can be carried out by any intelligent person, a special masseur not being essential. The surgeon himself should see that it is done as he wishes and that his instructions are not exceeded. After the first rubbing, the effects are so beneficial that the patient looks forward each day to its being performed. All that is necessary is to remove so much of the apparatus as will expose that part of the limb from which the œdematous swelling is to be expressed. To prevent chafing starch powder or French chalk should be used. Tapping, pinching, and other special forms of massage are not required. Throughout the course of after treatment constant daily care must be directed to the position of the foot. It is most important that this should be kept well flexed and that there should be no eversion of the sole. If it is desired to make use of plaster of Paris splints in the treatment of these fractures, this material should be so applied that it can be readily removed for purposes of massage. Croft's splint has such a purpose in view, and one of the most convenient materials for making this variety of splint especially suitable for private practice, on account of the rapidity and ease with which it can be applied, is Dr. Schwat Boa splint, which consists of layers of house flannel in a woven covering of white cotton impregnated with plaster of Paris, and ready for immediate use. After six weeks, splints can as a rule be altogether discarded, and the patient allowed about with crutches until seven or eight weeks have elapsed from the time of the injury. By exchanging a crutch for a stick, and giving up first the one stick and then the other, week by week, at the end of three months from the time of the accident, the patient should find himself perfectly strong, with a limb that is again quite sound.

